# 

**Published:** 1881-10-20

**Authors:** 


Entered at the Post-Office at Atlanta as second-class matter.

VOL. XI. OCTOBER 20, 1881. NO. 10

INDEX MEDICUS.

THE

### SOUTHERN MEDICAL RECORD:

S.G.O. INDEXED

### A MONTHLY JOURNAL OF PRACTICAL MEDICINE.

Terms: $2 00 per Annum, in Advance; 4 Copies $6.00; Single Copies 20 Cents.

*"QUICQUID PRÆCIPIES ESTO BREVIS."*

| | | | |
|---|---|---|---|
| William R. Warner & Co | 1 | Powers & Weightman, | 14 |
| Reed & Carnrick | 2–3 | N. Y. Pharmacal Association | 15 |
| J. L. Berg & Co | 4–5 | Geo. J. Howard & Bro | 16 |
| Powell's Combined Beef, etc | 6 | Trommer Extract of Malt | (ins. front cov.) |
| A. A. Mellier | 7 | Reed & Carnrick | (inside back cover.) |
| Sharp & Dohme | 8 | Parke, Davis & Co | (outside back cover.) |
| Mellin's Food | 9 | McKesson & Robbins | (colored leaves.) |
| Johnston's Fluid Beef | 9 | Parke, Davis & Co | (colored leaves.) |
| Thomas F. Goode | 10 | Scott's Emulsion | (colored leaves.) |
| O. C. St. Clair | 11 | Listerine | (colored leaves.) |
| Dundas, Dick & Co | 12 | Celerina | (colored leaves.) |
| Bedford Springs | 13 | Petrolina | (col. leaves) |
| Schumann | 14 | Southern Medical College | (col. leaves.) |

Address R. C. WORD, M.D., Managing Editor, Atlanta, Ga.

